# Prevalence and characteristics of Attention Deficit Hyperactivity Disorder in Adults with Autism Spectrum Disorders without intellectual disabilities

**DOI:** 10.1192/j.eurpsy.2025.750

**Published:** 2025-08-26

**Authors:** B. Demartini, M. Corbelli, F. Wiedemann, R. Faggioli, V. Nisticò

**Affiliations:** 1 Dipartimento di Salute Mentale e Dipendenze, ASST Santi Paolo e Carlo; 2 Department of Health Science, University of Milan, Milan, Italy

## Abstract

**Introduction:**

Autism spectrum disorder (ASD) and attention deficit hyperactivity disorder (ADHD) are two common neurodevelopmental conditions, whose prevalence in the general population has significantly increased in the last decade (2.2% and 2.5%, respectively). The co-occurring prevalence of ASD and ADHD is estimated at approximately 28% (Lai et al. 2019), and the differential diagnosis between these two conditions has become increasingly challenging, especially in adulthood. For instance, both individuals diagnosed with either ASD or ADHD might present social difficulties, despite the underlying causes are notably different: individuals with ASD struggle with social approach and communication, while individuals with ADHD might show distractibility and rapid loss of interest in social activities, or even exhibit behaviors perceived as annoying or rude, such as interrupting and intruding conversations (Antshel & Russo, 2019). Most importantly, in both ASD and ADHD (and especially in women) copying strategies such as the well-known “camouflaging” were observed, to mask autistic- or ADHD-related traits, to try to fit into a society mainly structured by and for neurotypical individuals (Lai & Baron-Cohen, 2015), but ultimately affecting their physical and mental health.

**Objectives:**

Aim of this study was to estimate the prevalence of ADHD traits and diagnosis in a sample of adult individuals with ASD without intellectual disabilities, examine sex differences in ADHD features, and explore the association between impulsivity and autistic traits.

**Methods:**

146 adults with ASD completed assessments for autistic-, ADHD-traits, and impulsivity. Those above the ADHD-traits cut-off underwent the Diagnostic Interview for ADHD in adults (DIVA-5).

**Results:**

42 subjects (28.8%) were diagnosed with ADHD comorbid with ASD (26 combined type, 16 inattentive, 0 impulsive). Most diagnosed subjects (71.4%) were females, but males scored higher on inattentive and hyperactive-impulsive symptoms. Autistic traits were positively correlated with attentive impulsiveness.

**Conclusions:**

Adults with ASD without intellectual disabilities show a significant prevalence of comorbid ADHD, particularly with inattentive symptoms. Attention difficulties are common in both disorders. Further studies and tailored diagnostic processes are needed to assess sub-threshold symptoms in ASD, ADHD, and other neurodevelopmental conditions.

**Disclosure of Interest:**

None Declared

